# Identification of CDCA2 as a Diagnostic and Prognostic Marker for Hepatocellular Carcinoma

**DOI:** 10.3389/fonc.2021.755814

**Published:** 2021-10-01

**Authors:** Zhenjun Yu, Yu Zhang, Shuai Shao, Qi Liu, Yuhan Li, Xiaoxiao Du, Kun Zhang, Mengxia Zhang, Haixia Yuan, Qiang Yuan, Tong Liu, Yingtang Gao, Yijun Wang, Wei Hong, Tao Han

**Affiliations:** ^1^ Department of Hepatology and Gastroenterology, The Third Central Clinical College of Tianjin Medical University, Tianjin, China; ^2^ Department of Histology and Embryology, School of Basic Medical Sciences, Tianjin Medical University, Tianjin, China; ^3^ Department of Hepatology and Gastroenterology, Tianjin Union Medical Center Affiliated to Nankai University, Tianjin, China; ^4^ Department of Hepatobiliary Surgery, The Tianjin Third Central Hospital, Tianjin, China; ^5^ Tianjin Key Laboratory of Extracorporeal Life Support for Critical Diseases, Artificial Cell Engineering Technology Research Center, Tianjin Institute of Hepatobiliary Disease, The Tianjin Third Central Hospital, Tianjin, China

**Keywords:** CDCA2, hepatocellular carcinoma, biomarker, prognosis, bioinformatics

## Abstract

**Objective:**

Hepatocellular carcinoma (HCC) is one of the most common and malignant tumors with an insidious onset, difficult early diagnosis, and limited therapy options, resulting in a poor prognosis. Cell division cycle associated 2 (CDCA2), also known as Repo-Man, plays an important role in regulating mitosis and DNA repair, but the involvement of CDCA2 in HCC remains unclear.

**Methods:**

The differentially expressed genes that were significantly upregulated in multiple RNA sequencing datasets of HCC were screened. Receiver operating characteristic (ROC) curve analysis was performed to identify diagnostic markers for HCC. Least absolute shrinkage and selection operator Cox regression analysis was performed to screen the prognosis-related genes. The screening and analyses identified CDCA2 as the target gene in this study. The expression of CDCA2 was analyzed in public databases and clinical specimens, and CDCA2 involvement in HCC was explored by both bioinformatic analysis and *in vitro* experiments.

**Results:**

The level of CDCA2 was enhanced in HCC compared with healthy livers. Overexpression of CDCA2 positively correlated with the pathological grade and TNM stage of the diseases. Furthermore, CDCA2 was found to be an independent prognostic predictor. An excellent prognostic model of HCC was successfully constructed with CDCA2 in combination with TNM stage. Bioinformatic analysis revealed that CDCA2 was closely associated with the cell cycle, apoptosis, and p53 signaling pathway. Silencing CDCA2 in Huh7 cells resulted in significant upregulation of p53 and the downstream PUMA and NOXA and a subsequently increased apoptosis. Inhibition of p53 signaling and apoptosis was found after overexpression of CDCA2 in L02 cells. Strikingly, the proliferation of cells was not affected by CDCA2.

**Conclusions:**

CDCA2 was a novel diagnostic marker for HCC, and overexpression of this gene reflected poor pathological grade, stage, and clinical prognosis. CDCA2 promoted the pathogenesis of HCC by suppressing the p53-PUMA/NOXA signaling and the subsequent apoptosis.

## Introduction

Primary liver cancer is the sixth most common and fourth most deadly cancer worldwide, and the incidence is on the rise in both developing and developed countries (1). In China, there are approximately 466,000 new cases of liver cancer each year, accounting for more than half of all new cases worldwide (2). Hepatocellular carcinoma (HCC) accounts for 75%–85% of primary liver cancers (1) and occurs mainly in patients with underlying liver diseases, such as hepatitis B or C virus infection or alcohol abuse, while the recent increase in non-alcoholic fatty liver disease, as well as metabolic syndrome and obesity, has further increased the risk of HCC (3). Due to the complex pathogenesis and insidious onset, most patients are already in advanced stages when diagnosed with HCC (4). Moreover, treatment options for HCC are limited and ineffective, resulting in a 5-year survival rate of 17%–53% and a recurrence rate of up to 70% of patients with early-stage HCC treated by surgical resection (5). These contribute to its persistently high mortality rate, with more than 600,000 deaths from HCC worldwide each year (6). Therefore, it is essential to find effective biomarkers for the diagnosis and prognosis of HCC.

A cancer biomarker is defined as a substance or process that indicates the presence of cancer. Many biomarkers of HCC have been reported in the literature, but only a few of them have been validated and successfully used in clinical practice (7). For example, alpha fetoprotein (AFP) is the most widely used surveillance indicator for HCC and represents the most cost-effective strategy; yet, up to 40% of patients with HCC, especially those still in the early stages, have normal AFP levels (8), leading to a growing debate about AFP as the routine surveillance. Bioinformatic analysis is more likely to overcome the shortcomings of known biomarkers because it allows researchers to deeply analyze comprehensive data from a large number of clinical samples from different independent studies, which provides a database for discovering promising biomarkers and updating our understanding of cancers (9).

The aim of this study was to find a potential diagnostic and prognostic marker for HCC and explore the biological functions through bioinformatic analysis. We have identified cell division cycle associated 2 (CDCA2) as our target gene and confirmed its overexpression in HCC tissues. CDCA2, also known as Recruits PP1 onto Mitotic chrominat anaphase (Repo-Man), plays an important role in the regulation of cell mitosis and DNA repair (10) and has also been found to be highly expressed in many different tumors. For example, the expression of CDCA2 is associated with massive size and the presence of lymph node metastasis in oral squamous cell carcinoma (11). Overexpressed CDCA2 promotes proliferation of colorectal cancer (CRC) cells by targeting upregulation of cyclin D1 (CCND1) through activation of the phosphoinositide 3-kinase (PI3K)/AKT pathway, while a specific PI3K inhibitor (LY294002) blocks the pro-proliferative effect of CDCA2 on CRC cells (12). In addition, high expression of CDCA2 has been reported in melanoma, clear cell renal cell carcinoma, and prostate cancer, and its upregulation was positively correlated with tumor progression (13–15). However, the expression, biological functions, and potential mechanisms of CDCA2 in HCC have rarely been reported.

## Materials and Methods

### Clinical Samples

A total of 87 patients with HCC diagnosed from 2015 to 2018 from the Third Central Hospital of Tianjin were collected, and the surgically resected HCC tissues and corresponding paracancerous tissues were frozen and stored in -80°C for further analysis.

### Analysis of Upregulated Differentially Expressed Genes

In the Gene Expression Omnibus (GEO) database, high-throughput RNA sequencing (RNA-seq) datasets from GSE136846, GSE104310, and GSE138485 were selected. The corresponding Sequence Read Archive data files were uncompressed to complete fastqc quality testing, and the Spliced Transcripts Alignment to a Reference (STAR) software (16) was used for accurate alignment to obtain the read counts of each gene. The R software (version 3.6) with edgeR package was applied to analyze the raw gene count data, including background correction, quantile normalization, and log2 conversion. Differentially expressed genes (DEGs) were screened under the condition of |log2FoldChange|>1, adjusted p-value <0.05, and the upregulated DEGs in all three datasets displayed an overlap region in the Venn diagram.

We obtained the RNA sequence data of liver HCC in The Cancer Genome Atlas (TCGA) database (https://www.cancer.gov) and the International Cancer Genome Consortium (ICGC) database (https://daco.icgc.org). RNA sequence data for healthy human liver and other organs were obtained in the Genotype-Tissue Expression (GTEx) database (https://gtexportal.org). Data of liver tissues from TCGA and GTEx were merged, and the batch effects were removed by processing with the removeBatchEffect function of R software with limma package. A total of 276 cases of healthy and paraneoplastic liver tissues and 371 cases of HCC tissues were subsequently included in this study. The edgeR package was used to normalize and process these data before log2 conversion.

### Least Absolute Shrinkage and Selection Operator Cox Regression and Receiver Operating Characteristic Curve Analysis

Least absolute shrinkage and selection operator (LASSO) Cox regression analysis between RNA levels of upregulated DEGs and survival data in 371 HCC cases was completed by R software with glmnet package to screen prognosis-related genes. Sensitivity and specificity of upregulated DEGs for the diagnosis of HCC were then evaluated by receiver operating characteristic (ROC) curve analysis in the combined GTEx and TCGA data. Therefore, CDCA2, a gene rarely reported in previous HCC studies, was identified as the target gene in this study. A 5-fold cross-validated logistic model was constructed using the scikit-learn package of python software (version 3.7) to further evaluate the effectiveness of CDCA2 in the diagnosis of HCC, and the Precision, Recall, Accuracy, and F1 score were used to evaluate the performance of this classification model.

### Correlation Analysis of CDCA2

In GTEx data, the expression levels of CDCA2 in various organs of healthy people were analyzed. The correlation of CDCA2 with clinical characteristics including gender, age, family history and clinical stage of HCC, and pathological grade was analyzed in TCGA data.

It reports that infiltrating stromal and immune cells play important roles in carcinogenesis. Assessment of stromal and immune cell infiltration scores in tumor tissues can be obtained using RNA expression data from TCGA samples. ESTIMATE, stromal, and immune scores for HCC samples were obtained in the ESTIMATE database (https://bioinformatics.mdanderson.org/estimate) and used to assess their correlations with CDCA2.

### Quantitative Real-Time Polymerase Chain Reaction

qPCR analysis was performed as described previously (17). Primer sequences were described as follows: hGAPDH: Forward: 5ʹ-ACCCAGAAGACTGTGGATGG-3ʹ, Reverse: 5ʹ-TTCAGCTCAGGGATGACCTT-3ʹ; hCDCA2: Forward: 5ʹ-TGCCGAATTACCTCCTAATCCT-3ʹ, Reverse: 5ʹ-TGCTCTACGGTTACTGTGGAAA-3ʹ; hP53: Forward: 5ʹ-GCTTTGAGGTGCGTGTTTGT-3ʹ, Reverse: 5ʹ-AGAGGAGCTGGTGTTGTTGG-3ʹ; hPUMA: Forward: 5ʹ-TGGGTGAGACCCAGTAAGGA-3ʹ, Reverse: 5ʹ-CCTGCTCTGGTTTGGTGAGT-3ʹ; hNOXA: Forward: 5ʹ-CGAAGATTACCGCTGGCCTA-3ʹ, Reverse: 5ʹ-TGAACTGTTTCTCCCCAGCC-3ʹ; hP21: Forward: 5ʹ-TGCCGAATTACCTCCTAATCCT-3ʹ, Reverse: 5ʹ-TGCTCTACGGTTACTGTGGAAA-3ʹ; hPCNA: Forward: 5ʹ-GGTTACTGAGGGCGAGAAGC-3ʹ, Reverse: 5ʹ-GACCGGCTGAGACTTGCGTA-3ʹ; hBAX: Forward: 5ʹ-TCAGGATGCGTCCACCAAGAA-3ʹ, Reverse: 5ʹ-TCTGCAGCTCCATGTTACTGTCCA-3ʹ; hBCL2: Forward: 5ʹ-GTGGAGAGCGTCAACCGGGAGA-3ʹ, Reverse: 5ʹ-GGGCCGTACAGTTCCACAAAGGC-3ʹ; hCTGF: 5ʹ-ATCCAGGCAAGTGCATTGGTA-3ʹ, Reverse: 5ʹ-GGGCCTCTTCTGCGATTTC-3ʹ; hCyclinD1: 5ʹ-TCAAGTGTGACCCGGACTG-3ʹ, Reverse: 5ʹ-ATGTCCACATCTCGCACGTC-3ʹ.

### Western Blot

Total proteins were extracted by cell lysis buffer radioi-mmunoprecipitation assay (RIPA; Cell Signal Technology, USA) with 1% phosphatase inhibitor and 1% phenylmethylsulfonyl fluoride (PMSF). Polyvinylidene fluoride (PVDF) membranes containing proteins separated on sodium dodecylsulfate–polyacrylamide gel electrophoresis (SDS-PAGE) gels were blocked with 5% nonfat milk and incubated with primary antibodies overnight at 4°C, and chemiluminescence was used for detection. Protein quantification was performed using ImageJ software.

### Immunohistochemistry

Specimens were fixed continuously in 10% formalin for 2 days, transferred to different concentrations of ethanol, and paraffin embedded for histopathological analysis. Sections (5 μm) were stained with hematoxylin and eosin (H&E) for histopathological observation. Afterward, selected sections were treated with antigen repair, 3% H_2_O_2_ treatment, and incubation in goat serum to block nonspecific immune responses; primary antibodies were incubated overnight at 4°C, and diaminobenzidine was used to observe reaction products and monitored by microscopy. Five random scenes from each preparation were analyzed morphologically, and the mean percentage of positive products was plotted.

### Cell Culture and Antibodies

The human hepatocellular cells Huh7, HepG2, and PLC were maintained in Dulbecco’s modified Eagle’s medium (DMEM, Invitrogen, Camarillo, CA, USA) supplemented with 10% fetal bovine serum (FBS; Gibco, Gaithersburg, MD, USA), penicillin (100 U/ml), and streptomycin (100 μg/ml). The human hepatic cell L02 (obtained from Merck Millipore) was cultured in Roswell Park Memorial Institute 1640 Medium (Gibco, Grand Island, NY, USA) supplemented with 10% FBS, penicillin (100 U/ml), and streptomycin (100 μg/ml). All cells were cultured at 37°C in an atmosphere containing 5% CO_2_. The antibodies for Western blot are as follows: IKK (Abcam, 178870), IκBα (Cell Signaling Technology, 4814), p-IκBα (Cell Signaling Technology, 9246), p65 (Santa Cruz Biotechnology, sc-8008), p-p65 (Cell Signaling Technology, 3033), PARP1 (Proteintech, 13371-1-AP), BCL-2 (Proteintech, 12789-1-AP), BAX (Abcam, ab32503), Caspase8 (Abcam, ab25901), Caspase3 (Cell Signaling Technology, 9662), cleaved Caspase3 (Cell Signaling Technology, 9661), NOXA (Santa Cruz Biotechnology, sc-56169), PUMA (Abcam, ab33906), murine double minute 2 (MDM2; Santa Cruz Biotechnology, sc-56154), p53 (Santa Cruz Biotechnology, sc-126), p-p53(S15) (Abcam, ab223868), PCNA (Cell Signaling Technology, 13110), CDCA2 (Cell Signaling Technology, 14976), GAPDH (Cell Signaling Technology, 5174), goat anti-mouse IgG (Abcam, ab97023), and goat anti-rabbit IgG (Abcam, ab97051). The antibodies for immunohistochemistry (IHC) are as follows: CDCA2 (Sigma, HPA030049) and CD34 (Abcam, ab81289).

### Construction of Weighted Gene Co-Expression Network Analysis

The GSE89377 dataset contains 107 samples and 10 groups with the healthy liver as control and experimental groups ranging from chronic hepatitis, cirrhosis, dysplastic nodules, and early HCC to G1, G2, and G3 grade of HCC. The characteristic genes of the module could be linked to the evolution course from chronic liver disease to HCC. Gene expression matrix of GSE89377 was constructed, and the top 25% of genes with the greatest variation in samples were selected as the input data for subsequent weighted gene co-expression network analysis (WGCNA) by the R software with WGCNA package. Outlier samples were detected and excluded by sample hierarchical clustering method, and suitable soft thresholds were selected to achieve scale-free networks. Then, through the construction of adjacency and topological overlap matrix (TOM) and the calculation of corresponding dissimilarity, gene dendrogram and module identification were accomplished with dynamic tree cut. The module eigengenes were then clustered, and highly similar modules were fused to calculate the correlation between module eigengenes and clinical groups. The module including CDCA2 was selected for further functional enrichment analysis.

### Single-Cell Sequencing Data Analysis

Single-cell RNA sequence data of HCC cells were obtained from the dataset of GSE124395. HCC cells were divided into different cell clusters, and the distribution of CDCA2 in them was analyzed by the R software with Seurat package. Gene enrichment analysis in the cell cluster with the highest expression level of CDCA2 was performed according to the marker genes, from which core genes were obtained by Cytoscape software with mcode package, and protein–protein interaction (PPI) network was mapped.

### Biological Function and Pathway Enrichment Analysis

Module eigengenes in WGCNA and marker genes of the cell cluster in single-cell sequencing data were analyzed by the R software with clusterprofiler package for Gene Ontology (GO) and Kyoto Encyclopedia of Genes and Genomes (KEGG) pathway analysis. In addition, TCGA samples were divided into two groups based on the median values of CDCA2, and gene set enrichment analysis (GSEA) was performed by the R software with GSEA package. The cutoff criteria for GSEA were nominal p-value <0.05 and false discovery rate <0.25.

### Transient Transfection and Construction of Lentiviral Plasmids

For gene knockdown analysis, small interfering (si) RNA targeting the CDCA2 sequence and negative control siRNA were obtained from GenePharma Biological Technology (Shanghai, China). Lipofectamine Max (Invitrogen, Carlsbad, CA, USA) was applied to transfect the siRNA into Huh7 cells. The target sequences were UUCUCCGAACGUGUCACGUTT (NC), CACCUGCCUUUCUAAAUAUTT (CDCA2-si1), and UGACAGACUUGACCAGAAATT (CDCA2-si2), respectively. The full-length cDNA of CDCA2 was amplified by PCR and ligated to the lentiviral shuttle vector pCCL.PPT.hPGK.IRES.eGFP/pre to construct the high-expression plasmid LV-CDCA2 and the empty plasmid LV-Control. pMD2.BSBG, pMDLg/pRRE, and pRSV-REV were used to construct the lentivirus in HEK-293T cells. Infectious lentiviral solution was collected at 36 and 60 h after transfection and filtered through 0.45-μm PVDF filter.

### Cell Counting Kit-8 Cell Proliferation Assay

Cell Counting Kit-8 (CCK8) Proliferation/Toxicity Assay Kit (Sigma-Aldrich, St. Louis, MO, USA) was applied to detect the proliferation level of cells. Here, 10 μl/well (96-well plate) of CCK solution was added to the cultured cells and incubated for 3 h at 37°C; then, the absorbance value at 450 nm was measured.

### Statistical Analysis

Continuous data were expressed as mean ± standard deviation and analyzed with the *t*-test, one-way ANOVA test. Diagnostic and prognostic marker screening was performed by ROC curve analysis, Cox regression analysis, and Kaplan–Meier (KM) survival analysis. Statistical analysis was performed using SPSS 22.0 software (IBM, Armonk, New York, NY, USA) and R software (Foundation for Statistical Computing, Vienna, Austria), and p < 0.05 was considered a significant difference.

## Results

### Identification of Upregulated Differentially Expressed Genes in Hepatocellular Carcinoma Datasets

**Figure 1:**
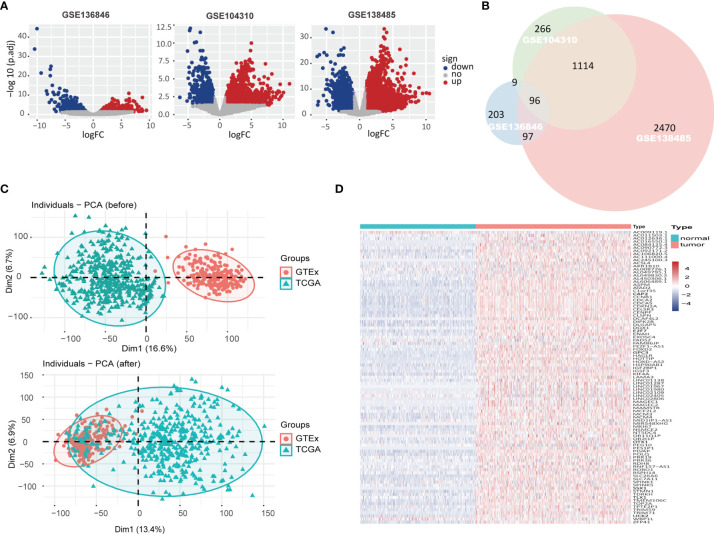
The upregulated differentially expressed genes (DEGs) in hepatocellular carcinoma (HCC) datasets. **(A)** Volcano plot showed gene expression in GSE136846, GSE104310, GSE138485 datasets with red/blue marker classification of upregulated/downregulated genes based on the criteria of |log2FoldChange| >1 and adjust p-value <0.05. **(B)** Venn diagram showed the overlap of significantly upregulated DEGs in GSE136846, GSE104310, and GSE138485 datasets. **(C)** Principal component analysis (PCA) illustrated the merging of Genotype-Tissue Expression (GTEx) and The Cancer Genome Atlas (TCGA) data before and after removal of batch effects. **(D)** In the merged GTEx and TCGA data, the expression matrix of 96 upregulated DEGs was subjected to unsupervised hierarchical clustering, with blue, red, and white representing lower, higher, and no expression differences between genes, respectively.

As shown in **Figure 1A**, 914 DEGs were identified in HCC datasets GSE136846, of which 405 genes were upregulated and 509 genes were downregulated, compared with the paracancerous tissue (**Supplementary Table S1**). Similarly, 2,480 DEGs were identified in GSE104310, with 1,485 upregulated genes and 995 downregulated genes, and 6,035 DEGs were identified in GSE138485, with 3,777 upregulated genes and 2,258 downregulated genes (**Supplementary Tables S2, S3**). A total of 96 overlapping upregulated DEGs were obtained from the above three GSE datasets as shown in the Venn diagram (**Figure 1B**). Considering that there were 371 HCC tissues and only 50 paracancerous tissues in TCGA, we obtained 226 healthy liver tissue data from the GTEx database. The principal component analysis (PCA) showed that direct merging of GTEx and TCGA data resulted in a significant batch effect (**Figure 1C_Up**); therefore, the limma package removeBatchEffect function was applied to process the merged data and obtained good results (**Figure 1C_Down**). Furthermore, gene expression profiles of the merged data confirmed the overexpression of these 96 genes in HCC tissues (**Figure 1D** and **Supplementary Table S4**).

### Identification of Key Genes Associated With the Diagnosis and Prognosis of Hepatocellular Carcinoma

**Figure 2:**
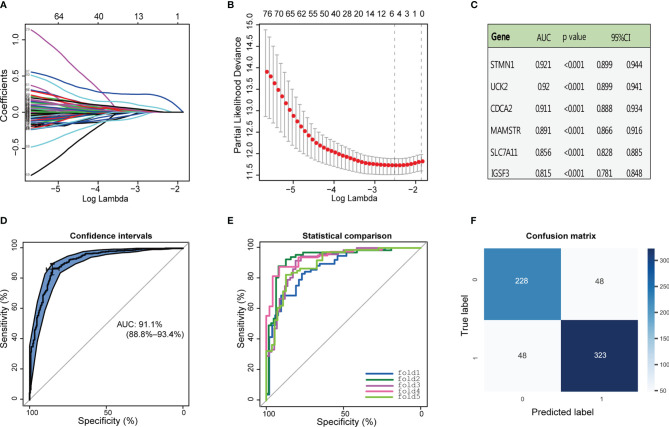
Identification of cell division cycle associated 2 (CDCA2). **(A, B)** Least absolute shrinkage and selection operator (LASSO) Cox regression analysis was performed with the expression profiles of 96 genes and survival information in The Cancer Genome Atlas (TCGA) cases, and six major prognosis-related genes were obtained. **(C)** The area under the curve (AUC), 95% CI, and p-values for the above six genes as diagnostic markers of hepatocellular carcinoma (HCC) were calculated in the combined Genotype-Tissue Expression (GTEx) and TCGA data. **(D)** Receiver operating characteristic (ROC) curve analysis was used to assess the sensitivity and specificity of CDCA2 as the marker gene of HCC. **(E)** Sensitivity and specificity regarding the diagnostic efficacy of CDCA2 for HCC in each fold data from the 5-fold cross-validation. **(F)** Confusion matrix showed that the model constructed with CDCA2 successfully predicted 228 healthy cases and 323 HCC cases.

ROC curve analysis was performed to evaluate the efficacy of the above 96 genes in the diagnosis of HCC in the combined GTEx and TCGA data, and 95% of these genes obtained area under the curve (AUC) values of 0.6 or higher (**Supplementary Table S5**). To identify DEGs that were also associated with HCC prognosis, LASSO Cox regression analysis of the 96 genes was completed with survival information from TCGA samples, and six prognosis-related genes were obtained (**Figures 2A, B**). The AUC and 95% CI values of these genes were demonstrated in **Figure 2C**. According to previous studies, a rarely reported gene, CDCA2, caught our attention. Although CDCA2 has been known to play an important role in cell cycle regulation, its function in HCC remains unclear. Therefore, CDCA2 was identified as the target gene in this study. ROC curve analysis in the merged data was used to assess the sensitivity and specificity of CDCA2 as a diagnostic biomarker for HCC with an AUC value of 0.911 (0.888–0.934) (**Figure 2D**). Afterward, a logistic model was developed to further assess the validity of CDCA2 for the diagnosis of HCC by a 5-fold cross-validation method (**Figure 2E**, **Supplementary Table S6**). A confusion matrix was built to obtain the mean values of Accuracy, Precision, Recall, and F1 score of 0.852, 0.871, 0.871, and 0.871, respectively (**Figure 2F**). These results suggested that CDCA2 had excellent discriminatory properties in classifying HCC from healthy liver tissues.

### Overexpression of CDCA2 Correlates With the Grade and Stage of Hepatocellular Carcinoma

**Figure 3:**
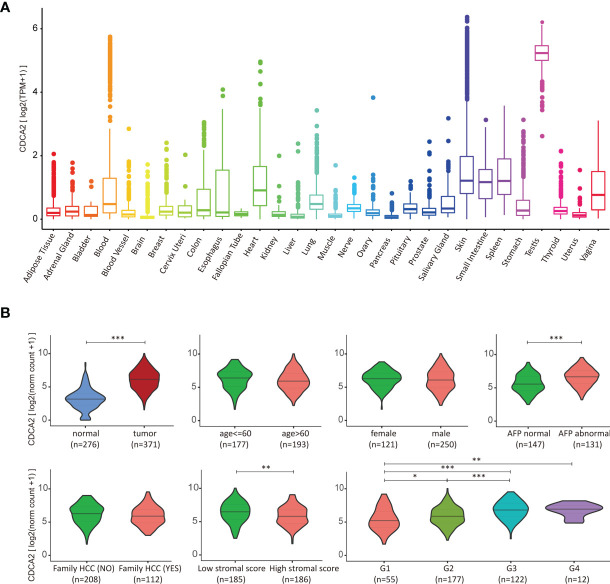
Cell division cycle associated 2 (CDCA2) correlated with hepatocellular carcinoma (HCC). **(A)** Gene expression of CDCA2 in normal human organs from the Genotype-Tissue Expression (GTEx) database. **(B)** Correlation analysis of CDCA2 with clinical indicators in The Cancer Genome Atlas (TCGA) cases and ESTIMATE database. *p < 0.05, **p < 0.01, ***p < 0.001.

The expression of CDCA2 in healthy human organs was obtained from the GTEx database, which showed that CDCA2 was overexpressed in testicle and down-expressed in other organs, especially in the brain, liver, and muscle (**Figure 3A** and **Supplementary Table S7**). Based on the evidence that CDCA2 was significantly increased in HCC tissues, we analyzed the correlation between clinical indicators and CDCA2 expressions in TCGA cases to further evaluate the role of CDCA2 in HCC. As shown in **Figure 3B**, CDCA2 was positively correlated with AFP level, pathological grade, and TNM stage, while there was no significant correlation with age, gender, and family history. The immune infiltration correlation analysis showed a significant negative correlation between CDCA2 and the stromal score, except the immune and ESTIMATE score (**Figure 3B** and **Supplementary Tables S8–S10**). These results suggested that CDCA2 was closely associated with the progression of HCC but weakly associated with the immune environment.

**Figure 4:**
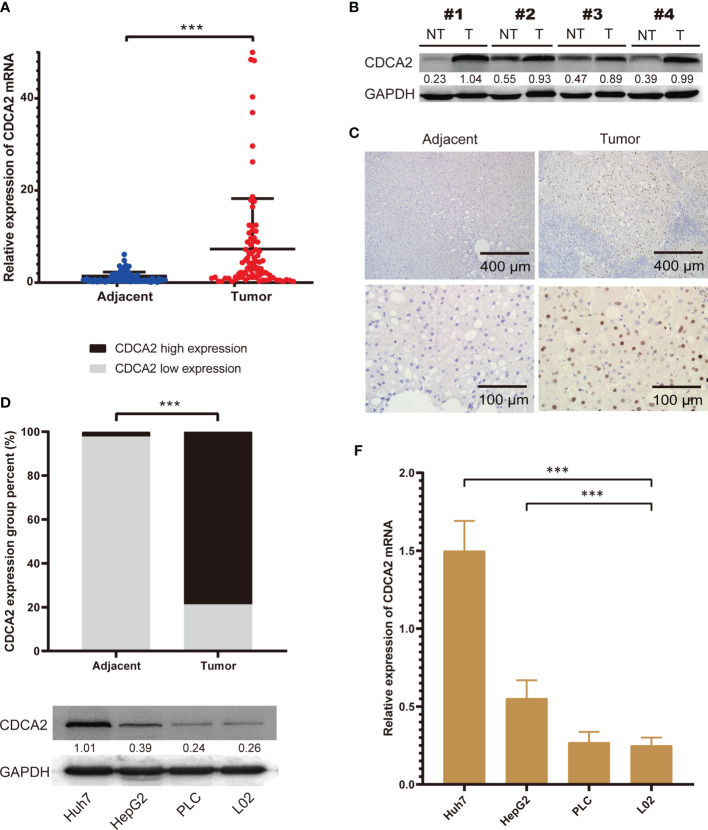
The expression of cell division cycle associated 2 (CDCA2) in hepatocellular carcinoma (HCC) tissues and cells. **(A)** The mRNA levels of CDCA2 in hepatocellular carcinoma (HCC) and paraneoplastic tissues in patients. **(B)** Protein levels of CDCA2 in four pairs of HCC and paraneoplastic tissues; quantitative analysis was performed with ImageJ software. **(C)** Immunohistochemistry (IHC) staining showed that CDCA2 was expressed in the nucleus of HCC cells. **(D)** Morphological analysis showed a positive average rate of 78.8% for CDCA2 in HCC and 2.3% in paraneoplastic tissues. **(E)** Protein levels of CDCA2 in Huh7, HepG, PLC, and L02 cells; quantitative analysis was performed with ImageJ software. **(F)** The mRNA levels of CDCA2 in four cells. ***p < 0.001.

To further confirm the expression of CDCA2 in HCC, we collected surgically resected HCC and corresponding paraneoplastic normal tissues from 87 patients, and the patients’ clinical characteristics were shown in **Supplementary Table S11**. Total RNA was extracted, and the results of qPCR showed that the mRNA level of CDCA2 in HCC was significantly enhanced than that in paracancerous tissues (**Figure 4A**). We detected the protein levels of CDCA2 in four pairs of HCC and paraneoplastic tissues, and Western blot showed that CDCA2 was significantly increased in HCC (**Figure 4B**). In addition, IHC staining showed an overexpression of CDCA2 in HCC compared with the paraneoplastic tissues (**Figures 4C, D**). H&E staining and IHC of CD34 were also performed (**Supplementary Figure S1**). Moreover, we examined CDCA2 expression in Huh7, HepG2, PLC, and L02 cells, the results showed that mRNA and protein levels of CDCA2 were relatively highest in Huh7 and lowest in PLC and L02 (**Figures 4E, F**).

### The Role of CDCA2 in Assessing the Prognosis of Hepatocellular Carcinoma

**Figure 5:**
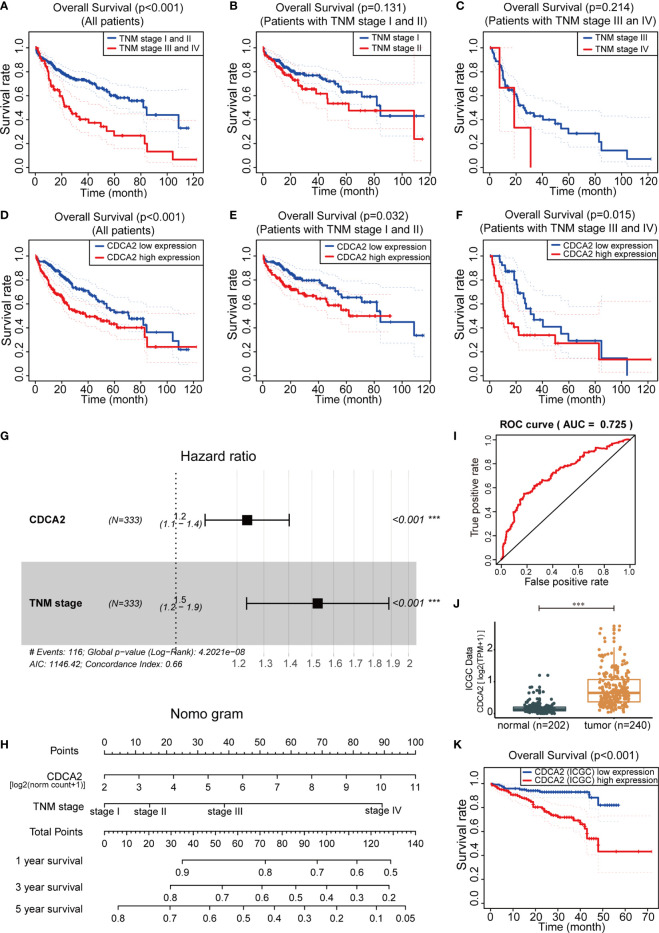
Cell division cycle associated 2 (CDCA2) was associated with hepatocellular carcinoma (HCC) prognosis. **(A–F)** In The Cancer Genome Atlas (TCGA) cases, the impact of TNM stage and CDCA2 on the prognosis of all HCC cases, early HCC cases (TNM stage I/II), and mid- to late-stage HCC cases (TNM stage III/IV) was analyzed by Kaplan-Meier (KM) survival. **(G)** Forest plot illustrated the hazard ratio (HR) values of CDCA2 and TNM stage by multivariate Cox regression analysis. **(H)** The nomogram of prognostic 1-, 3-, and 5-year survival rates for HCC was constructed based on CDCA2 expression values [log2 (norm count + 1)] and TNM stage. **(I)** Receiver operating characteristic (ROC) curve analysis of predictive efficacy of the nomogram model. **(J, K)** Comparison of mRNA levels and survival analysis of CDCA2 in HCC and paraneoplastic samples originating from Japan in International Cancer Genome Consortium (ICGC) database. ***p < 0.001.

The influence of clinical indicators and CDCA2 on the prognosis of HCC was analyzed by KM survival in TCGA cases. The prognosis-related indicators were the TNM stage and CDCA2 (p < 0.001) (**Figures 5A, D**), while other indicators including age, gender, pathological grade, family history, body mass index (BMI), and AFP were all not significantly correlated with HCC prognosis (**Supplementary Table S12**). Moreover, the differences in survival time could not be well distinguished by applying TNM stage in the early HCC group (TNM stage I/II) or in the mid- to late-stage HCC group (TNM stage III/IV) (p > 0.05) (**Figures 5B, C**); however, CDCA2 could distinguish well the survival time of these two groups (p < 0.05) (**Figures 5E, F**). Multivariate Cox regression analysis showed that both CDCA2 and TNM stage were independent HCC prognostic factors with hazard ratio (HR) values of 1.237 and 1.526, respectively (**Figure 5G**). Combining the two parameters to construct a nomogram, patient scores and corresponding 1-, 3-, and 5-year survival rates could be found according to CDCA2 level and TNM stage (**Figure 5H**). The predictive efficacy of the model was good, with an AUC value of 0.725, as analyzed by the ROC curve (**Figure 5I**). In addition, RNA data and corresponding survival information of 240 HCC cases and 202 paraneoplastic samples originating from Japan were obtained through the ICGC database (**Supplementary Tables S13, S14**). Consistent with our results, CDCA2 levels were significantly increased in HCC than in paraneoplastic tissues (**Figure 5J**). The efficacy AUC value of CDCA2 as a diagnostic marker for HCC was 0.925 (**Supplementary Figure S2**), and CDA2 was also shown to be significantly associated with HCC prognosis (p < 0.001) (**Figure 5K**).

### Construction of Weighted Gene Co-Expression Network Analysis and Identification of the Module Containing CDCA2

**Figure 6:**
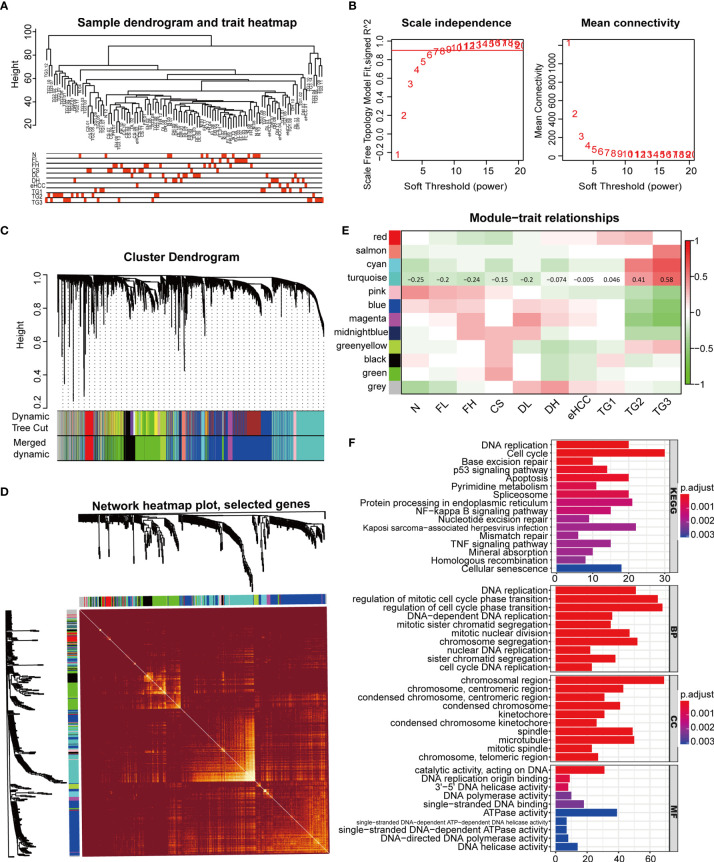
Weighted gene co-expression network analysis (WGCNA) construction and identification of the module containing cell division cycle associated 2 (CDCA2). **(A)** The WGCNA with group information was constructed through the RNA level of 107 samples in GSE89377, including normal liver (N), chronic hepatitis with low grade and high grade (FL and FH), cirrhosis (CS), dysplastic nodules with low grade and high grade (DL and DH), early-stage HCC (eHCC), and G1, G2, and G3 grade HCC (TG1, TG2, and TG3). **(B)** Analysis of scale-free fit index and the mean connectivity for various soft-thresholding powers. Testing the scale-free topology when β  =  9. **(C)** Hierarchical clustered expression maps of different genes based on topological overlap, where modules were branches of cluster trees. **(D)** Heat map of gene topological overlap matrix (TOM) overlap visualization with bright or dark colors representing higher or lower overlap, respectively. **(E)** In the correlation plot between module genes and clinical groups, each row corresponds to a module, columns represent clinical groups, red represents positive correlation, and green represents negative correlation. The correlation coefficients of the turquoise module were labeled in the figure. **(F)** Gene enrichment analysis of Gene Ontology (GO) and Kyoto Encyclopedia of Genes and Genomes (KEGG) signaling pathway was completed by R software.

The WGCNA with group information was constructed through the RNA data in GSE89377 (**Figure 6A**). In this study, we set the fit index to 0.9, corresponding to a power-weighted index of β = 9, to construct a scale-free network (**Figure 6B**). Gene clustering was performed based on TOM overlap, and 11 gene co-expression modules were identified after eliminating gray modules by dynamic tree cut (**Figure 6C**). The best clustering of genes in black, cyan, and turquoise green modules was found in the TOM overlap visualization heat map (**Figure 6D**), suggesting that the biological functions of genes related to these three modules were highly correlated. The target gene of this study, CDCA2, was located in the turquoise gene module, which was positively correlated with the pathogenesis and progression of HCC in the correlation plot (**Figure 6E** and **Supplementary Table S15**). KEGG pathway analysis in the turquoise module showed that these genes were closely associated with DNA replication, cell cycle, apoptosis, p53, and Nuclear Factor-kappa B (NF-κB) signaling. GO analysis showed that these genes were abundantly expressed in DNA replication, regulation of mitotic cell cycle, chromosome segregation, nuclear division, telomere maintenance, etc. (**Figure 6F**).

### CDCA2 Was Closely Related to the p53 Signaling Pathway and the Expression of P53 Gene

**Figure 7:**
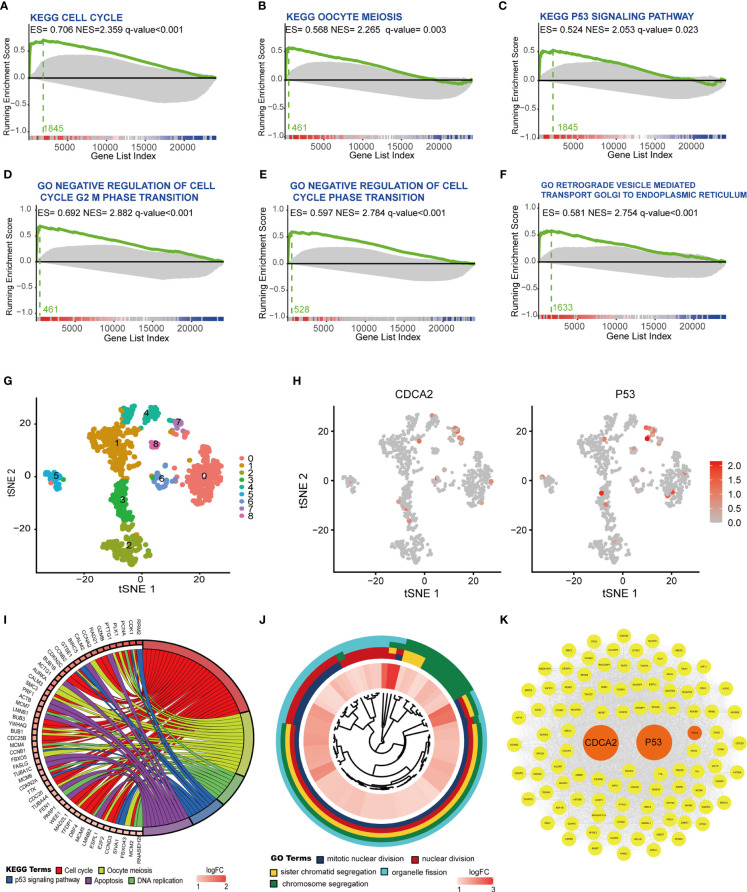
Cell division cycle associated 2 (CDCA2) was associated with the p53 signaling and the expression of P53. **(A–F)** Analysis of CDCA2 correlation with Kyoto Encyclopedia of Genes and Genomes (KEGG) and Gene Ontology (GO) gene sets was performed by the R software with gene set enrichment analysis (GSEA) package in The Cancer Genome Atlas (TCGA) database. Enrichment score (ES), normalized enrichment score (NES), and q values were shown in each vignette with the Zero cross at 13357. **(G)** Hepatocellular carcinoma (HCC) cells were delineated into nine clusters, from 0 to 8, shown in different colors with the R software with Seurat package, respectively. **(H)** Application of red to highlight the distribution of CDCA2 and p53 expression in different clusters in the tSNE map. **(I, J)** KEGG pathway and GO enrichment analysis of marker genes in cluster 7. **(K)** Protein–protein interaction (PPI) relationship of core genes associated with CDCA2 and p53.

The relationship of CDCA2 with KEGG and GO gene sets was further analyzed in TCGA by the R software with GSEA package. The results showed that the cell cycle, oocyte meiosis, and p53 signaling in the KEGG gene sets (**Figures 7A–C**) and the cell cycle regulation-related gene set in GO were closely related to CDCA2 (**Figures 7D–F**). Single-cell sequencing RNA data of HCC cells were obtained from the GSE124395 dataset. Data from three HCC cases with a total of 4,032 cells were normalized and filtered to >1,000 genes/cell, and 914 cells were identified as usable sequencing data. Dividing all cells into nine clusters by the R software with Seurat package based on the expressions of marker genes (**Figure 7G** and **Supplementary Figure S3**), the distribution of CDCA2 and P53 in HCC cells was highly consistent, and both were predominantly expressed in cells in cluster 7 (**Figure 7H**, **Supplementary Figure S4** and **Supplementary Tables S16, S17**). KEGG pathway analysis of cluster 7 cells with a total of 329 marker genes showed gene enrichment in cell cycle, DNA replication, oocyte mitosis, p53 signaling pathway, and apoptosis (**Figure 7I**). GO analysis showed gene enrichment in mitosis, nuclear division, etc. (**Figure 7J**), which were generally consistent with the WGCNA module gene enrichment and GSEA. Cytoscape software with mcode module was used to analyze the 100 core genes from the marker genes of cluster 7, and the PPI network was completed to demonstrate the mutual regulation of the core genes (**Figure 7K**).

### CDCA2 Inhibited Apoptosis Through p53 Signaling Pathway

**Figure 8:**
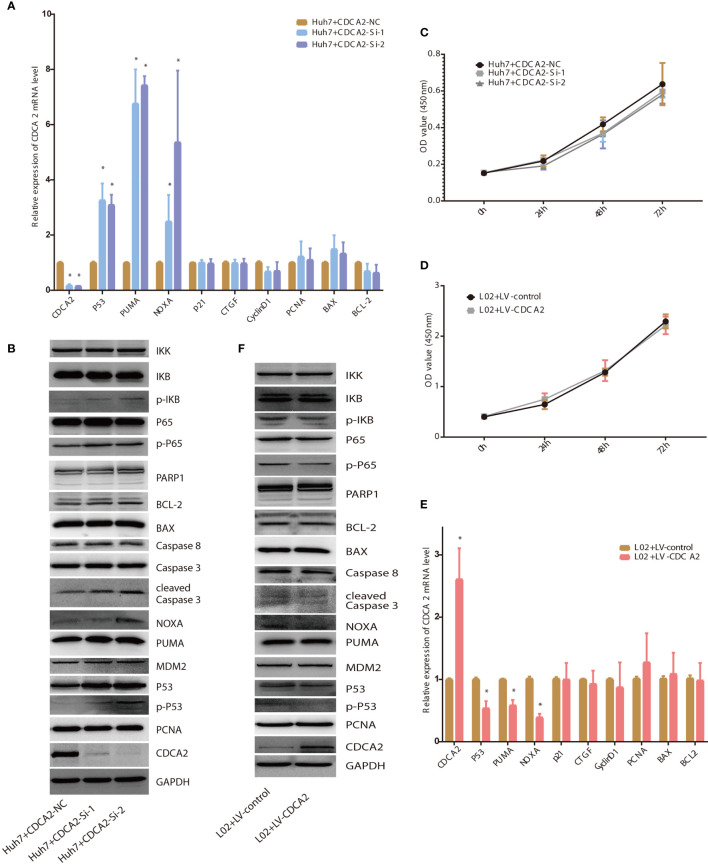
Cell division cycle associated 2 (CDCA2) foundations *via* p53. **(A, B)**. The mRNA and protein levels of the proliferation- and apoptosis-related genes in Huh7 treated with CDCA2 Si-1/2. **(C, D)** The proliferation of CDCA2 Si-1/2-treated Huh7 cells and LV-CDCA2-treated L02 cells at different time points detected by Cell Counting Kit-8 (CCK8) assay. **(E, F)** The mRNA and protein levels of the proliferation- and apoptosis-related genes in L02 treated with LV-CDCA2. *p < 0.05.

To verify the above results, CDCA2 was silently expressed in the Huh7. The mRNA levels of CDCA2 in Huh7 treated with CDCA2 Si-1/2 were significantly downregulated compared with those of the NC-treated group, while the mRNA levels of p53 and downstream genes PUMA and NOXA were significantly upregulated. The mRNA levels of genes related to cell cycle and proliferation including p21, connective tissue growth factor (CTGF), CyclinD1, and proliferating cell nuclear antigen (PCNA) were not significantly changed (**Figure 8A**). Western blot showed that the protein levels of CDCA2 treated with CDCA2 Si-1/2 were significantly downregulated compared with those of the NC group, and the levels of p53, phosphorylated p53 (p-p53), and the downstream target proteins, PUMA and NOXA, controlling apoptosis, and apoptosis-related protein cleaved-caspase3 were significantly upregulated (**Figure 8B**), suggesting that silencing CDCA2 promoted apoptosis through activation of the p53-PUMA/NOXA signaling pathway. However, levels of p53 upstream protein MDM2 and other apoptosis-related proteins including poly(ADP-ribose) polymerase 1 (PARP1), total Caspase3, Caspase8, BAX, BCL-2, and proliferation-related protein PCNA were not significantly changed (**Figure 8B**). CCK8 cell proliferation assay showed no significant differences in cell proliferation levels of Huh7 treated with CDCA2 Si-1/2 at 24, 48, and 72 h compared to those of the NC group (**Figure 8C**). Moreover, we treated L02 with lentivirus overexpressing CDCA2, and both qPCR and Western blot showed, in CDCA2-overexpressed cells, p-p53, NOXA, and cleaved-Caspase3 were significantly downregulated (**Figures 8E, F** **)**, while there were no significant changes in MDM2, PCNA, and other apoptosis-related protein levels (**Figure 8F**). CCK8 cell proliferation assay showed no significant differences in the proliferation levels of L02 treated with LV-CDCA2 at 24, 48, and 72 h (**Figure 8D**). In addition, as a result of bioinformatic analysis, we also confirmed the regulatory role of CDCA2 on NF-κB signaling pathway. Silent expression of CDCA2 resulted in significant upregulation of p-p65 and p-IKB, while overexpression of CDCA2 resulted in significant downregulation of p-p65 and p-IKB (**Figures 8B, F** **)**. However, the consequence of CDCA2 regulating NF-κB signaling remains to be further investigated.

## Discussion

CDCA2 belongs to the family of cell division cycle associated proteins (CDCAs), which are critical to tumor progression in a growing body of evidence. Oral squamous cell carcinoma tissues tend to overexpress CDCA1 and CDCA3, which may prevent cellular G1 phase block by inhibiting cyclin-dependent kinase inhibitors (18). CDCA3, CDCA5, and CDCA8 are overexpressed and function as oncogenes in HCC and gastric and breast cancer (19, 20). CDCA2 is also a nuclear protein that binds to protein phosphatase 1 (PP1) (10). Previous studies have shown that CDCA2/PP1 regulates mitotic processes, including chromatin remodeling, nuclear membrane reorganization, and DNA repair (21). In addition, CDCA2/PP1 regulates the formation of heterochromatin and dephosphorylates H3S28, and it is necessary and sufficient for heterochromatin protein 1 binding and H3K27me3 recruitment (22). Recently, more studies have focused on the role of CDCA2 in tumorigenesis development; for instance, CDCA2 was shown to be upregulated in clear cell renal cell carcinoma tissues, and the magnitude of upregulation correlated with the degree of malignancy (13). CDCA2 was also found to exacerbate the malignant progression of melanoma by upregulating the proliferation and migration capacity of the tumor cells (14). A recent publication reported that CDCA2, CDCA3, CDCA5, and CDCA8 were upregulated in HCC, which might have potential diagnostic and prognostic values for HCC. However, extensive bioinformatic analyses, enlarged clinical samples, and detailed research are needed to further confirm the result and reveal the underlying mechanism of CDCAs (23).

In this study, we have firstly screened 96 DEGs that were significantly upregulated in several RNA-seq datasets of HCC and then screened prognosis-related genes by LASSO Cox regression, identifying CDCA2 as the target gene. The analyses of GTEx, TCGA, and ICGC data revealed that CDCA2 was overexpressed in HCC, compared with most healthy organs, with high sensitivity and specificity for the diagnosis of this carcinoma. The overexpression of CDCA2 in HCC was also confirmed with clinical samples. Further analysis demonstrated that CDCA2 was positively correlated with AFP, pathological grade, and TNM stage of HCC. Moreover, CDCA2 was shown to be an independent prognostic factor for HCC, and the prognostic model constructed in combination with TNM stage obtained a good predictive efficacy. Our finding is consistent with a previous study related to prostate cancer, which indicates that CDCA2 is positively correlated with the histological grade, clinical stage, and prognosis (15).

To investigate the mechanism of CDCA2 involvement in HCC, we performed WGCNA, GSEA of TCGA, and KEGG and GO analysis of single-cell sequencing data, all of which showed that CDCA2 was closely related to cell cycle, apoptosis, and p53 signaling pathway. Silencing of CDCA2 in Huh7 resulted in significant upregulation of p53 and the downstream PUMA and NOXA and an increased apoptosis. However, no significant changes were found in PCNA, cyclin D1, as well as the levels of cell proliferation at different time points. Consistently, LV-CDCA2 treatment in L02 showed that the p53 signaling and apoptosis were inhibited, with no significant changes in proliferation. As an important tumor suppressor, p53 is involved in a variety of biological processes, including classical functions such as cell cycle arrest, apoptosis, and cellular senescence (24). Moreover, p53 also plays an important regulatory role in tumor metastasis, cell metabolism, autophagy, and iron death (25). Both Puma and Noxa, direct targets of p53, which encode proapoptotic proteins containing the BH3 structural domain, and their overexpression lead to rapid apoptotic cell death (26). In this study, CDCA2 inhibited apoptosis of HCC cells by inhibiting the p53-PUMA/NOXA pathway; however, we failed to find a regulatory effect of CDCA2 on the proliferation of Huh7 and L02. Since cell cycle is a multifactorial, highly ordered and precisely regulated life process, the dysregulation of cell cycle is the root cause of uncontrolled tumor cell growth. P53 directly stimulates the expression of p2l, a suppressor of cell cycle protein-dependent kinase, and inhibits the mitotic G1 to S phase transition (27). In this study, we found extremely low mRNA levels of p21 in Huh7 and L02, and p21 protein could not be detected before or after the treatment for CDCA2 intervention. Whether this could explain the fact that CDCA2 has no influence on proliferation needs further investigation.

In addition, bioinformatic analysis and *in vitro* cellular assays showed that CDCA2 significantly inhibited the signaling of NF-κB, which is a pleiotropic transcription factor that regulates inflammation, natural immunity, cell survival, and proliferation and is a crucial link between inflammation and tumor (28). Since the role of NF-κB in HCC is paradoxical (29), some studies have shown that NF-κB promotes inflammation-associated cancers (30), while inactivation of liver-specific NF-κB promotes the development and progression of HCC, suggesting that NF-κB may act as a tumor suppressor (31, 32). In an oncogene MYC-induced HCC model, researchers specifically established liver-specific deletion of NF-κB essential modulator (NEMO) mice and found that NEMO deletion accelerated tumor progression and shortened survival. Moreover, deletion of NEMO modified the tumor phenotype from HCC to combined hepatocellular cholangiocarcinoma, further aggravating the aggressiveness and heterogeneity of the tumor (33). In this study, CDCA2 may simultaneously promote the development of HCC by inhibiting NF-κB signaling pathway, but the exact mechanism is unclear.

## Conclusion

In conclusion, we found that CDCA2 was a new diagnostic marker for HCC through bioinformatic analysis and clinical samples; overexpression of CDCA2 reflected poor pathological grading, staging, and clinical prognosis of HCC patients. The pathogenesis of HCC was promoted by CDCA2 in *in vitro* experiments, which inhibited apoptosis by suppressing the p53-PUMA/NOXA signaling pathway. Therefore, CDCA2 is expected to be applied as a potential target for the clinical diagnosis and therapy of HCC in the future.

## Data Availability Statement

The raw data supporting the conclusions of this article will be made available by the authors without undue reservation.

## Ethics Statement

The studies involving human participants were reviewed and approved by the Research Ethics Committee of Tianjin Third Central Hospital. The patients/participants provided their written informed consent to participate in this study. Written informed consent was obtained from the individual(s) for the publication of any potentially identifiable images or data included in this article.

## Author Contributions

ZY and YZ designed the study and drafted the final manuscript. ZY, YZ, SS, QL, YL, XD, and MZ performed the experiments. ZY and KZ analyzed the data. HY, QY, TL, and YG helped to collect samples. TH, YW, and WH conceived the study and revised the final manuscript. All authors contributed to the article and approved the submitted version.

## Funding

This work was supported by the National Natural Science Foundation of China (No. 81870429, 81670558, and 81800542).

## Conflict of Interest

The authors declare that the research was conducted in the absence of any commercial or financial relationships that could be construed as a potential conflict of interest.

## Publisher’s Note

All claims expressed in this article are solely those of the authors and do not necessarily represent those of their affiliated organizations, or those of the publisher, the editors and the reviewers. Any product that may be evaluated in this article, or claim that may be made by its manufacturer, is not guaranteed or endorsed by the publisher.

## Abbrevations

HCC, hepatocellular carcinoma; AFP, alpha fetoprotein; CDCA2, cell division cycle associated 2; Repo-Man, Recruits PP1 onto Mitotic chrominat anaphase; CRC, colorectal cancer; DEGs, differentially expressed genes; GEO, Gene Expression Omnibus; STAR, Spliced Transcripts Alignment to a Reference; TCGA, The Cancer Genome Atlas; ICGC, International Cancer Genome Consortium; GTEx, Genotype-Tissue Expression; LASSO, least absolute shrinkage and selection operator; ROC curve, receiver operating characteristic curve; qPCR, quantitative real-time polymerase chain reaction; IHC, immunohistochemistry; H&E, hematoxylin and eosin; WGCNA, weighted gene co-expression network analysis; TOM, topological overlap matrix; GO, Gene Ontology; KEGG, Kyoto Encyclopedia of Genes and Genomes; GSEA, gene set enrichment analysis; siRNA, small interfering RNA; CCK8, Cell Counting Kit-8; PCA, principal component analysis; AUC, area under the curve; CDCAs, cell division cycle associated proteins; NEMO, NF-κB essential modulator.
