# Supplementary figures and images for: Identification of CDCA2 as a Diagnostic and Prognostic Marker for Hepatocellular Carcinoma
Source: Front Oncol. 2021 Oct 1;11:755814. doi: 10.3389/fonc.2021.755814 (PMC8517522; doi:10.3389/fonc.2021.755814)

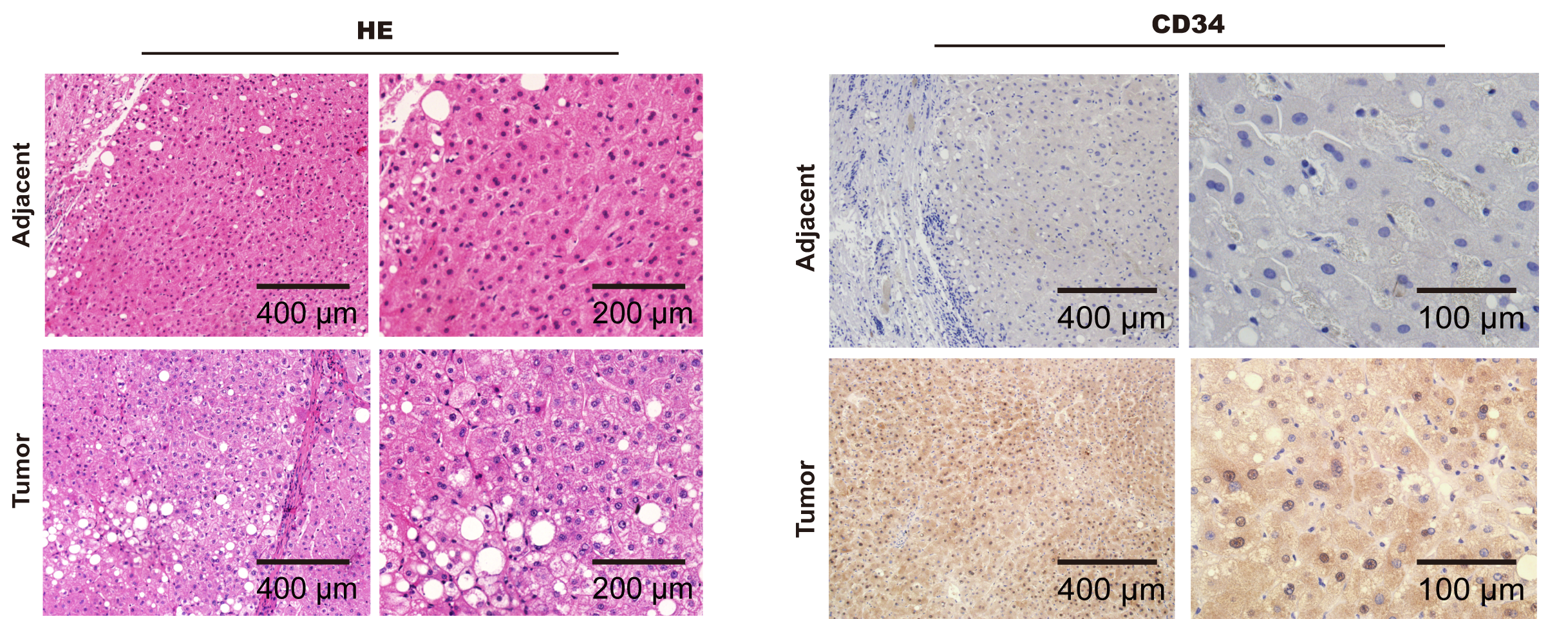

Supplement: Supplementary file 1 [file DataSheet_1.zip › Supplementary Figure S1.tif]

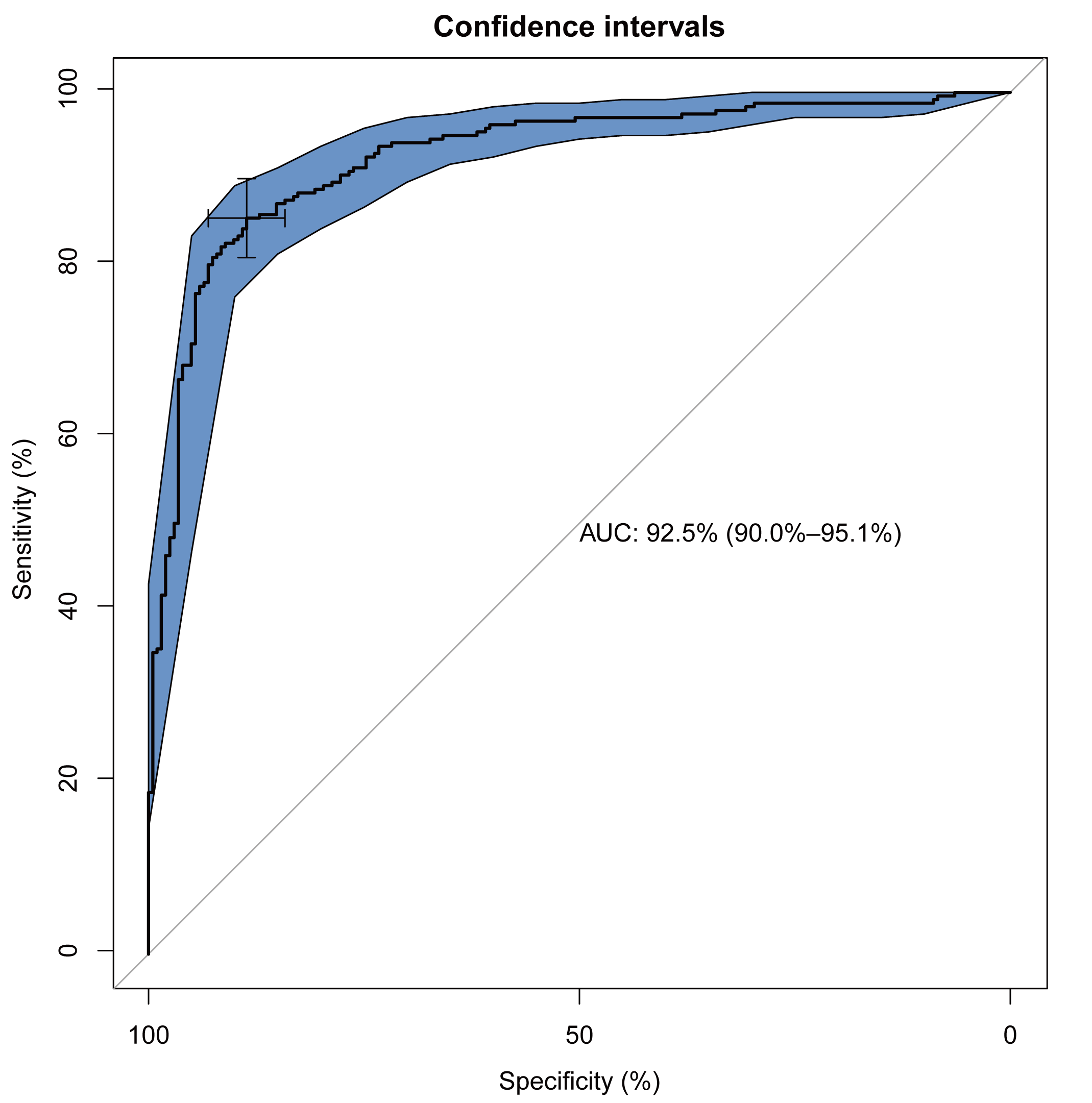

Supplement: Supplementary file 1 [file DataSheet_1.zip › Supplementary Figure S2.tif]

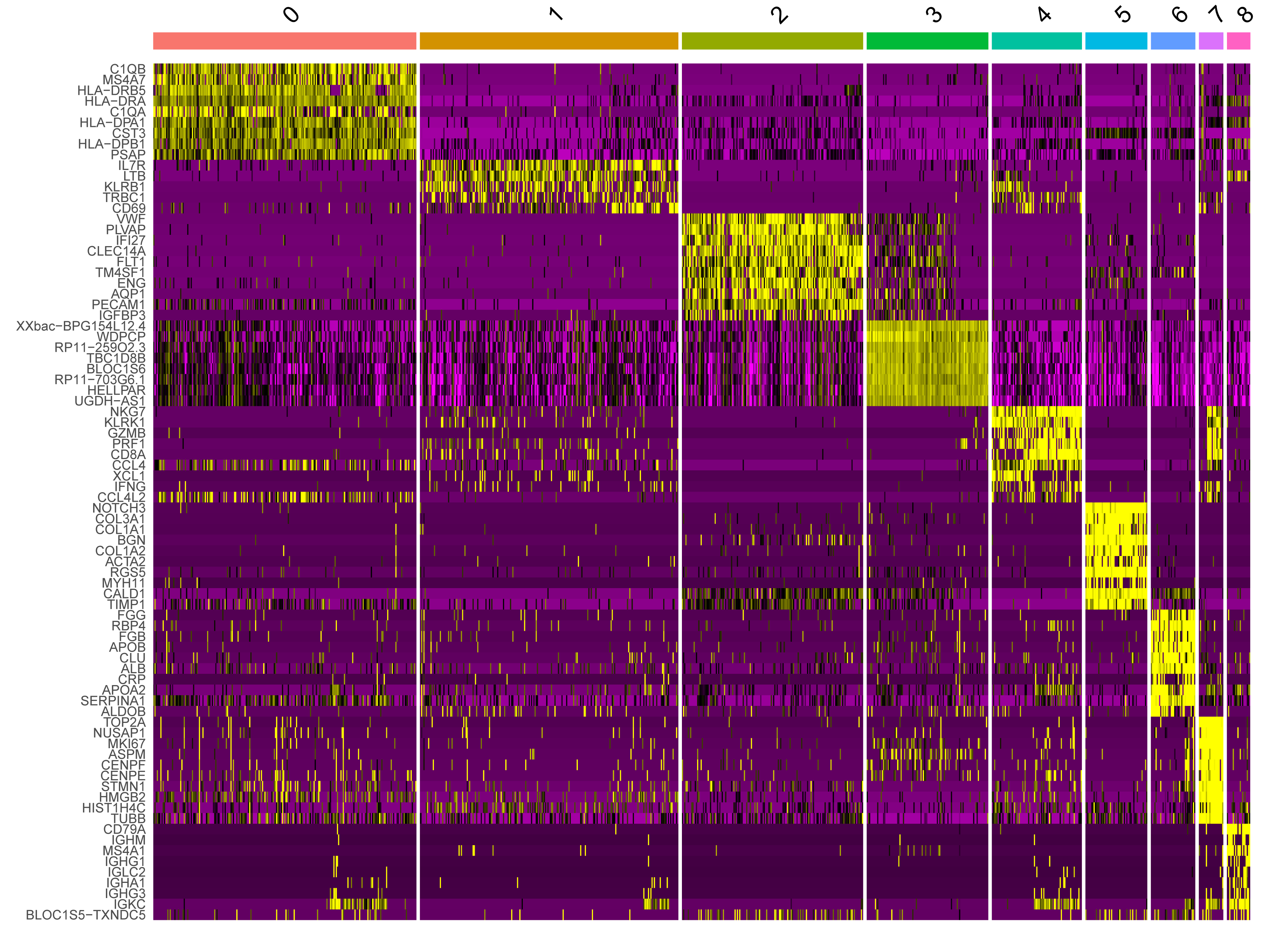

Supplement: Supplementary file 1 [file DataSheet_1.zip › Supplementary Figure S3.tif]

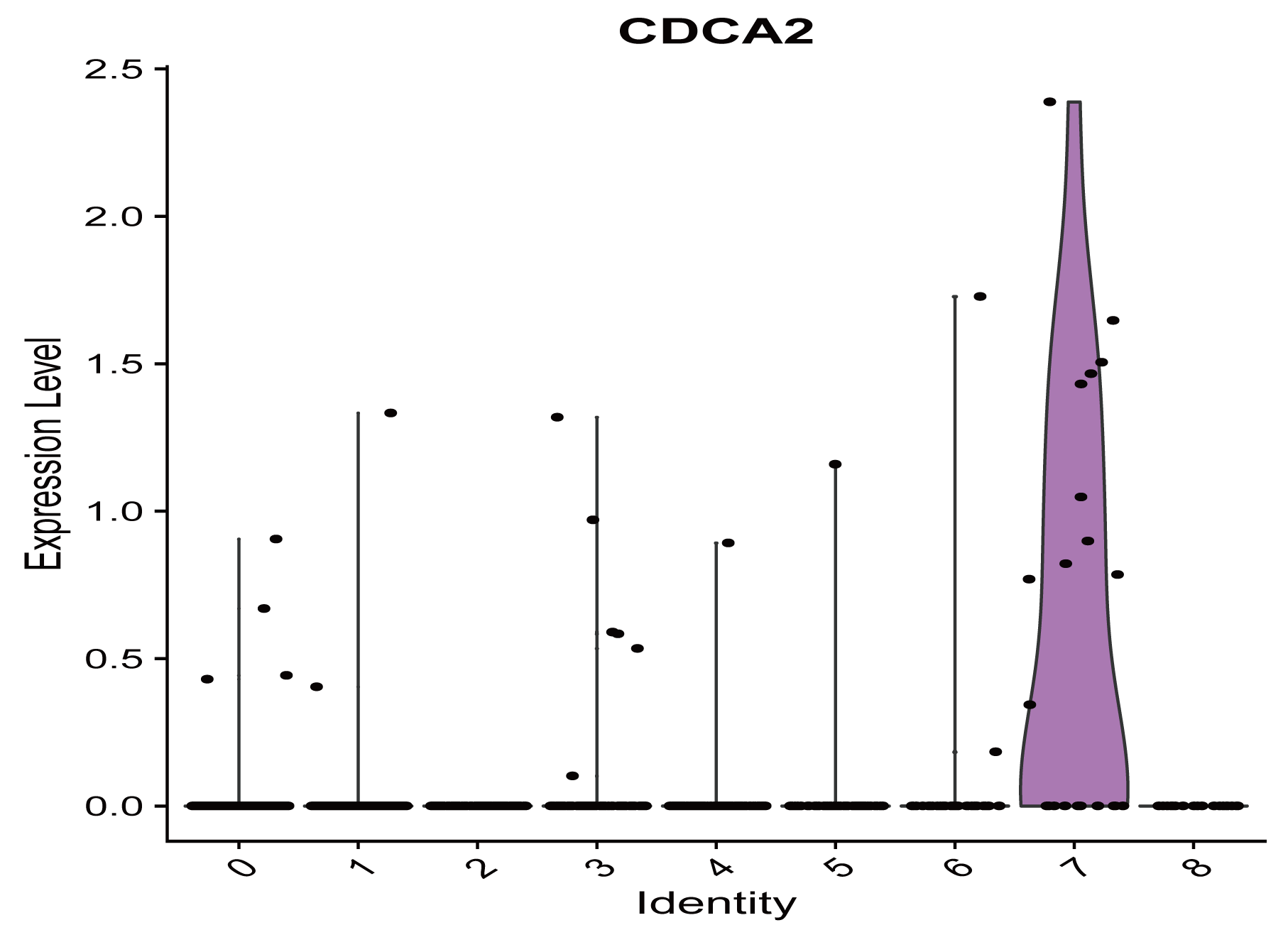

Supplement: Supplementary file 1 [file DataSheet_1.zip › Supplementary Figure S4.tif]
